# Poor sleep quality indirectly contributes to higher sexual risk-taking by increasing the likelihood of engaging in substance use among LGBTQ+ individuals

**DOI:** 10.3389/fpsyt.2025.1613882

**Published:** 2025-06-18

**Authors:** Efe Sarı, Nevin Durdu, Beril Ay, Barış Sancak

**Affiliations:** ^1^ Acibadem Mehmet Ali Aydinlar University School of Medicine, Istanbul, Türkiye; ^2^ Department of Psychiatry, Acibadem Mehmet Ali Aydinlar University School of Medicine, Istanbul, Türkiye

**Keywords:** sleep, LGBTQ+, substance, risk-taking, sexuality, health, chemsex

## Abstract

**Background:**

Poor sleep quality (PSQ) is disproportionately prevalent among LGBTQ+ individuals and has been linked to substance use, sexual dysfunction and sexual risk-taking (SRT). However, the interplay between sleep health, substance use, SRT, and sexual dysfunction remains underexplored in diverse LGBTQ+ populations. This study investigates whether substance use mediates the relationship between PSQ and SRT among LGBTQ+ individuals in Turkey.

**Methods:**

A cross-sectional survey, using snowball sampling was conducted among 249 LGBTQ+ individuals in Turkey. Measures included the Pittsburgh Sleep Quality Index (PSQI), Index of Sexual Risk-Taking (ISRT), Arizona Sexual Experiences Scale (ASEX), AUDIT-C for alcohol use, and self-reported recent substance use. Logistic and linear regression analyses were used to assess associations between PSQ, substance use, and SRT. Mediation analysis was conducted using structural equation modeling (SEM).

**Results:**

PSQ was highly prevalent (80.7%) and significantly associated with alcohol use and chemsex-related substance use (CRSU). CRSU was strongly linked to increased SRT (β = 1.489, p <.001) and served as a significant mediator in the PSQ-SRT relationship (β = 1.045, p = .047). No significant mediation effect was found for alcohol use.

**Conclusion:**

Poor sleep quality indirectly contributes to higher sexual risk-taking among LGBTQ+ individuals by increasing the likelihood of engaging in chemsex-related substance use. These findings highlight the need for integrated public health interventions addressing sleep health, substance use, and sexual risk-taking in LGBTQ+ communities, particularly in settings with rising substance use rates.

## 1 Introduction

Sleep is essential to healthy living, and poor sleep quality (PSQ) is well known to have detrimental effects on physical and mental well-being (1). Individuals with PSQ consistently report higher levels of health risk factors and have a higher risk of all-cause mortality (2–5).

Disproportionately higher rates of PSQ have been reported by lesbian, gay, bisexual, transgender, and queer (LGBTQ+) individuals (6, 7) and there remains a considerable gap in our understanding of this disparity. To date, various risk factors have been identified to explain this discrepancy, such as financial hardship, depression, stress, using tobacco, alcohol, and substances (6, 7). By gathering insights, more effective sleep healthcare strategies tailored to the needs of LGBTQ+ individuals can be developed (7, 8).

PSQ has been associated with substance use, and individuals seeking help for sleep disorders are more likely to be users of illicit substances and alcohol (9, 10). Previous research highlights that this relationship between substance use and PSQ might be bidirectional in nature. Chronic sleep disturbances may lead to increased substance use, presumably due to impaired decision-making skills, and substances used can further delay sleep onset and create a vicious circle (11–13).

Sleep deprivation, substance use, and sexual activity predominantly occur at night, suggesting a possible interrelation between the three (14, 15). PSQ has been linked to increased risk-taking behavior, further reinforcing the possibility that PSQ, substance use, and sexual risk taking (SRT) may amplify the risk associated with each other (14). PSQ is particularly associated with increased SRT, and substance use among men who have sex with men (MSM) (16). Additionally, sleep deprivation and tiredness are known to increase sexual desire, potentially leading to increased sexual activity and SRT during nighttime hours (17). All these findings reinforce the idea that substance use and SRT may be significantly associated with PSQ.

Previous research indicates that the initial use of substances is often driven by the desire to enhance sexual pleasure, encourage disinhibition, and facilitate lengthy sex sessions while maintaining wakefulness; however, long-term use has been associated with sexual dysfunction (18, 19). This phenomenon where individuals use drugs to intensify sexual activity, chemsex, is observed in 4% to 41% of the MSM community (18). Despite this, there remains a significant gap in the literature regarding sexual dysfunction among sexual minorities and women (20). Sexual dysfunction is particularly important in the context of sleep and substance use research since PSQ is well known correlate with sexual dysfunction (21). Considering the bidirectional relationship between substance use and PSQ, this worsening vicious cycle may be a compounding risk factor that is responsible for sexual dysfunction and SRT among people who report PSQ.

To the best of our knowledge, no study has yet examined the relationship of PSQ with substance use and SRT in any sexual and gender minorities other than MSM. Existing research on women’s sleep health consistently shows that women experience poorer sleep. Sexual minority women face more sleep disturbances than heterosexual women, men, and sexual minority men (7). Moreover, transgender individuals may be at the highest risk for PSQ among LGBTQ+ individuals (22). Given the disparities experienced by sexual minority women and gender minorities, it is crucial to examine this relationship between substance use, SRT, and PSQ among all LGBTQ+ individuals. As such, the hypotheses that were investigated in this study are:

1. LGBTQ+ individuals who report poorer sleep have a higher likelihood of using substance and alcohol, engaging in sexual risk taking, and experiencing sexual dysfunction.
2. LGBTQ+ individuals who report substance use and/or alcohol have a higher likelihood of engaging in sexual risk taking and experiencing sexual dysfunction.
3. Substance use and/or alcohol consumption mediates the relationship between poor sleep quality and both sexual risk taking and sexual dysfunction among LGBTQ+ individuals.

## 2 Methodology

### 2.1 Design and participants

The sample (N=249) for this cross-sectional study was recruited using an anonymous online questionnaire. The form link was distributed via social media platforms (‘WhatsApp’, ‘Instagram’, ‘Twitter/X’), using snowball sampling method. The study was introduced to potential participants via flyers and descriptive texts, which stated its aim as investigating the relationships between alcohol and drug use, sleep behavior, and sexual practices among LGBTQ+ individuals. 251 participants completed the survey of whom 2 were excluded. Exclusion criteria for those cases were not filling out the survey completely and not self-identifying as LGBTQ+. Answers for the form were accepted between 29.8.2024 and 29.9.2024. The questionnaire was repeatedly shared on different social media platforms and over the e-mail accounts of LGBTQ+ organizations, to reach a diverse study population. Inclusion criteria were as follows: (i) self-identifying as LGBTQ+, (ii) being at least 18 years old, (iii) living in Turkey, and (iv) reading and writing in Turkish.

### 2.2 Measures

The survey included demographic questionnaire to gather information on participants age (years), living arrangement (in a dormitory, with my spouse/partner/family, alone), being out or closeted about their LGBTQ+ identity, and regular usage of dating apps. Questions about working arrangements included education status, employment status, if working in shifts (full time mornings, full/part time evenings), and monthly income (no regular income, ranging from the minimum wage to more than three times the current minimum wage).

Participants were asked to indicate which gender they identify the most from the following options: cis-woman, cis-man, trans-man/transmasculine nonbinary, trans-woman/transfeminine nonbinary, and nonbinary/genderfluid/genderqueer. Additionally, they were asked to indicate their sexual orientation, with options including heterosexual, gay or lesbian, bisexual/pansexual/queer, and asexual. Cisgender participants were required to complete the Arizona Sexual Experience Scale (ASEX). Participants who engage in penetrative sex were asked about their sexual role (always insertive, mostly insertive, both insertive and receptive, mostly receptive, and always receptive).

To measure substance use, drug categories were classified into: chemsex-related substances (amphetamine, methylphenidate, methamphetamine, cocaine, MDMA, LSD, GHB, ketamine, magic mushroom), depressants (sleep medicines, opioids, heroin, benzodiazepines), and cannabinoids (Marijuana/Cannabis/THC). Participants were asked to indicate all the drugs they had used without a prescription or outside of their prescribed medical purpose within the last 3 months. The question was close-ended and presented in a multiple-choice format.

Alcohol use was measured using the AUDIT-C questionnaire, scored between 0 and 12, with higher scores indicating greater consumption (23). A score of 3+ in women, transgender, and gender-expansive participants, and 4+ in men, was considered positive for high alcohol use (24).

Pittsburgh Sleep Quality Index (PSQI) is a self rated questionnaire, that evaluates sleep quality and disturbances over the past month using 24 questions (likert type and open ended questions). The component scores are summed to generate a global score ranging from 0 to 21, with higher scores indicating poorer sleep quality. A score below 5 indicates good sleep quality (25). The PSQI was validated in 1996 in a Turkish sample, demonstrating reliable clinical and clinimetric properties over a 12-month period in healthy individuals, patients with depression, and those with sleep disorders, supporting its use in both clinical practice and psychiatric research (26).

The Index of Sexual Risk-Taking (ISRT) measures an individual’s engagement in risky sexual behaviors over the past 12 months and throughout their lifetime (27). The score ranges between 0 and 10, with higher scores indicating more risky sexual behaviors (28). Following consultations with the developer of the index and feedback from participants, particularly gender-diverse and lesbian women participants, three items related to condom use and anal sex were excluded to enhance validity. The modified version demonstrated a Cronbach’s alpha of 0.67. Certain ISRT questions are only relevant to participants whose sexual orientation matches descriptions on the Kinsey Scale; therefore, only these participants were included in the sample. Consequently, 25 participants were excluded in analyses involving ISRT.

Psychological and physiological arousal, orgasm, feelings of satisfaction, and sexual urges were measured using Arizona Sexual Experiences Scale (ASEX) (29). Comprising of 6 questions, the total score ranges between 5 and 30 (30). Lower scores indicate stronger and easier arousability and more satisfying sexual responses, while higher scores suggest sexual dysfunction. As certain questions on the ASEX specifically address biological sex functions and are designed exclusively for cis-male and cis-female individuals, only cisgender participants were asked to complete this section to ensure reliability and avoid potential distress among gender-diverse participants. Therefore, 102 participants were excluded in analyses involving ASEX.

### 2.3 Statistical analysis

All analyses were conducted using IBM SPSS version 22, except for the mediation analysis, which was performed using R version 4.4.0. Characteristics of the sample data were represented with mean values, standard deviation (SD), and frequencies.

Bivariate logistic regression analyses were performed to examine the association between the PSQ and all predictor variables. Multivariable logistic regression analyses were conducted to examine the association between PSQ and predictor variables AUDIT-C, chemsex-related substance use (CRSU), ISRT, and ASEX, while adjusting for living arrangement, employment, monthly income, gender, and sexual orientation. Additionally, linear regression analyses were conducted to evaluate associations between CRSU, AUDIT-C, and ISRT, and bivariate logistic regression analyses were performed to assess the relationships between CRSU, AUDIT-C, and ASEX. Odds ratios (ORs) for bivariate analyses, adjusted odds ratios (aORs) for multivariable models, and 95% confidence intervals (CIs) are reported for all relevant findings.

The mediating effect was evaluated using structural equation modeling (SEM) to decompose the total and indirect effects of the mediator. The mediation analyses were conducted in R version 4.4.0 using the lavaan package (31). The model specified three pathways (a, b, and c) to investigate the effect of the mediator (M) in the relationship between exposure (X) and outcome (Y). Pathway a assessed the relationship between X and M, while controlling for covariates including monthly income, employment status, living arrangement, gender, and sexual orientation. Pathway b evaluated the association between M and Y, with the same covariates. Pathway c (direct effect) analyzed the relationship between X and Y, adjusting for M and all covariates. The indirect effect, representing the mediation pathway through M, was calculated as a×b. The total effect was derived as the sum of the direct and indirect effects (c+a×b). The model was estimated using a weighted least squares (WLS) estimator. Statistical significance was set at α=0.05.

### 2.4 Ethics

Participation to the survey was anonymous. Each participant was asked to read and digitally sign an informed consent form before answering the survey. Ethics approval was granted by ATADEK-’Acıbadem Mehmet Ali Aydınlar University Ethics committee’ (number 2024-12/526).

## 3 Results

### 3.1 Descriptive statistics

The final cohort comprised 249 adult individuals who identified as LGBTQ+ and living in Turkey. The mean age was 25 years (SD = 5.89), with a range of 18 to 50 years. Transgender and nonbinary individuals constituted 40.1% of the cohort, while cisgender participants accounted for 59.8%. Regarding sexual orientation, 51.4% identified as bisexual, 40.2% as gay, 4.8% as heterosexual, and 3.6% as asexual.

For education and socioeconomic status, 49.4% of participants were students, and 91.6% had received an undergraduate-level education or higher. Present employment was reported by 39.0%, with most employed participants (68.1%) working morning shifts only. 68.3% had a stable monthly income, but 42.6% earned less than twice the minimum wage (MW). During the data collection period, the MW was 17,002 Turkish Liras (TRY), equivalent to approximately 500 USD.

Regarding social and lifestyle factors, 32.5% of participants lived with their families, and 26.5% lived alone. 70.7% identified as closeted in their daily lives. Regular use of dating applications and alcohol use disorder were each reported by 40.6% of participants. Substance use was indicated by 37.3% of the cohort. The most used substance was marijuana (21.3%), followed by chemsex-related substances (18.9%).

Scaled-based outcomes revealed that 25.9% of participants experienced sexual dysfunction, while 80.7% reported poor sleep quality. The mean ISRT score was 3.0 (SD = 1.85), with a range of 0 to 7. Detailed cohort characteristics are presented in **Table 1**.

**Table 1:** Demographic characteristics of the cohort.

Characteristics	Frequency (%)
Living Arrangement	
Partner	30 (12.0)
Alone	66 (26.5)
Family	81 (32.5)
Friends	37 (14.9)
Dormitory	35 (14.1)
Education	
Postgraduate	41 (16.5)
University	187 (75.1)
High school	21 (8.4)
Employment	
Employed	97 (39.0)
Unemployed	29 (11.6)
Student	123 (49.4)
Working Hours	
Only mornings	109 (68.1)
Also working nights	51 (31.9)
Monthly Income	
Above 3 MW	41 (16.4)
2-3 MW	23 (9.2)
1-2 MW	50 (20.1)
0-1 MW	56 (22.5)
Unstable	79 (31.7)
Out/Closeted	
Out	73 (29.3)
Closeted	176 (70.7)
Regular Dating App Use	
No	148 (59.4)
Yes	101 (40.6)
Alcohol Use Disorder (AUDIT-C)	
No	101 (40.6)
Yes	148 (59.4)
Substance Use	
No	156 (62.7)
Yes	93 (37.3)
Day/Night person	
Morning	113 (45.4)
Night	136 (54.6)
Gender	
Cis-man	72 (28.9)
Cis-woman	77 (30.9)
Nonbinary	67 (26.9)
Trans-man	20 (8)
Trans-woman	13 (5.2)
Sexual Orientation	
Gay/Lesbian	100 (40.2)
Asexual	9 (3.6)
Heterosexual	12 (4.8)
Bisexual	128 (51.4)
Sexual Position	
Top	24 (10.2)
Vers Top	38 (16.2)
Vers	96 (40.8)
Bottom	30 (12.8)
Vers Bottom	47 (20)
ASEX	
No	109 (74.1)
Yes	38 (25.9)
PSQI	
No	48 (19.3)
Yes	201 (80.7)

MW, Minimum wage.

### 3.2 Regression analyses

#### 3.2.1 Poor sleep health (hypothesis 1)

Bivariate logistic regression analyses were conducted to examine the association between PSQ and its predictor variables. The results in detail are depicted in **Table 2**. The analyses revealed that age, living arrangement, employment, working hours, and monthly income were significantly associated with sleep quality. Being younger and identifying as a “night person” were associated with PSQ. Individuals living in a dormitory or with friends also had increased odds of reporting PSQ compared to those living with their partner.

**Table 2:** Factors associated with poor sleep quality among LGBTQ+ individuals.

Variables	% Poor sleep	Bivariate, OR (95% CI)
Age		
18-50	80.7	0.94** (0.89-0.98)
Living arrangement		
Dormitory	91.4	6.17** (1.52-24.97)
Alone	78.8	2.15 (0.83-5.52)
Family	80.2	2.35. (0.93-5.91)
Friends	89.2	4.77** (1.33-17.10)
Partner (ref.)	63.3	–
Education		
High school	85.7	2.20 (0.54-8.95)
University	81.8	1.65 (0.75-3.61)
Postgraduate (ref.)	73.2	–
Employment		
Student	85.4	2.02* (1.03-3.98)
Unemployed	82.8	1.66 (0.57-4.83)
Employed (ref.)	74.2	
Working hours		
Full/part time evenings	90.2	3.49** (1.26-9.63)
Full time mornings (ref.)	72.5	–
Monthly income		
Unstable	83.5	3.59** (1.52-8.49)
0-1 MW	91.1	7.22*** (2.38-21.89)
1-2 MW	88	5.19** (1.80-14.92)
2-3 MW	69.6	1.61 (0.54-4.78)
Above 3 MW (ref.)	58.5	–
Out/Closeted		
Closeted	83	1.59 (0.82-3.08)
Out (ref.)	75.3	–
Dating app use		
Yes	85.1	1.64 (0.84-3.21)
No (ref.)	77.7	–
Day/Night person		
Night	85.3	1.91* (1.00-3.61)
Day (ref.)	75.2	–
Gender		
Nonbinary	83.6	1.34 (0.56-3.16)
Trans-woman	69.2	0.59 (0.16-2.19)
Trans-man	80	1.05 (0.30-3.61)
Cis-woman	81.8	1.18 (0.52-2.66)
Cis-man (ref.)	79.2	–
Sexual orientation		
Asexual	100	*perfect divider*
Heterosexual	50	0.31 (0.09-1.07)
Bisexual+	85.9	1.93. (0.98-3.80)
Gay/Lesbian (ref.)	76	–
Sexual position		
Bottom	76.7	1.64 (0.49-5.44)
Vers bottom	89.4	4.20* (1.19-14.76)
Vers	83.3	2.50. (0.91-6.82)
Vers top	76.3	1.61 (0.52-4.99)
Top (ref.)	66.7	–
AUDIT-C		
Yes	87.2	2.73** (1.43-5.21)
No (ref.)	71.3	–
Depressant substances^a^		
Yes	94.6	4.84* (1.12-20.92)
No (ref.)	78.3	–
Chemsex-related substances^b^		
Yes	91.5	2.99* (1.01-8.79)
No (ref.)	78.2	–
Weed		
Yes	84.9	1.44 (0.63-3.30)
No (ref.)	79.6	–
ISRT		
0-7	80.7	1.23** (1.02-1.49)
ASEX		
Yes	92.1	3.65* (1.03-12.86)
No (ref.)	76.1	–

CI, confidence interval; OR, odds ratio.

a Includes sleep medicines, opioids, heroin, benzodiazepines.

b Includes amphetamine, methylphenidate, methamphetamine, cocaine, MDMA, LSD, GHB, ketamine, magic mushroom.

.p<0.1, *p<0.05, **p<0.01, ***p<0.001. Statistically significant values are shown in bold.

Regarding employment, individuals working full-time or part-time evening shifts had increased odds of reporting PSQ compared to those working full-time morning shifts. Students had increased odds of reporting PSQ compared to currently employed individuals. Monthly income also influenced sleep quality; participants with unstable income or income below two MW reported more PSQ compared to those earning more than three MW.

Alcohol use and the use of chemsex-related or depressant substances were associated with PSQ, while marijuana use showed no significant association. Higher SRT scores were associated with PSQ. Furthermore, individuals reporting sexual dysfunction had higher odds of reporting PSQ.

Sleep quality was not associated with gender, sexual orientation, being closeted or out, level of education, or the use of dating apps. Regarding sexual position, individuals reporting engagement in the “vers bottom” position had higher odds of reporting PSQ.

In the multivariable logistic regression analyses, CRSU (aOR: 3.47; 95% CI [1.03-11.68], p<.05) and alcohol use (aOR: 2.86; 95% CI [1.36-6.00]; p<.01) were significantly associated with PSQ after controlling for covariables living arrangement, employment, monthly income, gender, and sexual orientation (Model 1). However, after adding ISRT and ASEX to the model only alcohol use (aOR: 3.21; 95% CI [1.17-8.77]; p<.05) was significantly associated with poor sleep (Model 3). Also, Model 1 was significant (p<.001) whereas the Model 3 was not (p=0.78). In all models, all covariables had Variance Inflation Factor (VIF) lower than 2. The sample size of model 3 was 138 due to exclusions regarding ISRT and ASEX while the sample size of model 1 was 249. Further information can be found in **Supplementary Material 1**.

#### 3.2.2 Sexual risk taking and dysfunction (hypothesis 2)

A linear regression analysis was conducted to examine the association between alcohol consumption and SRT. The model was statistically significant (p=.031, R^2^=.021). The regression coefficient for AUDIT-C was β=0.545 (95% CI [0.050, 1.041], p=.031). Similarly, the model for the association between CRSU and SRT was statistically significant (p<.001, R^2^=.102). The regression coefficient for CRSU was β=1.489 (95% CI [0.905, 2.074], p<.001), indicating that participation in CRSU is associated with an average increase of 1.489 points in ISRT scores.

The logistic regression model assessing the association between alcohol consumption and sexual dysfunction was not statistically significant (p=.752, R^2^ = 0.001), indicating that AUDIT-C did not significantly predict ASEX (OR=1.12, 95% CI [0.53,2.36]). However, the model for the association between CRSU and sexual dysfunction was significant (p=.021, R^2^ = 0.102) and CRSU was associated with decreased odds of sexual dysfunction (OR=0.09, 95% CI [0.01, 0.69]).

### 3.3 Mediation analysis (hypothesis 3)

**Figure 1:**
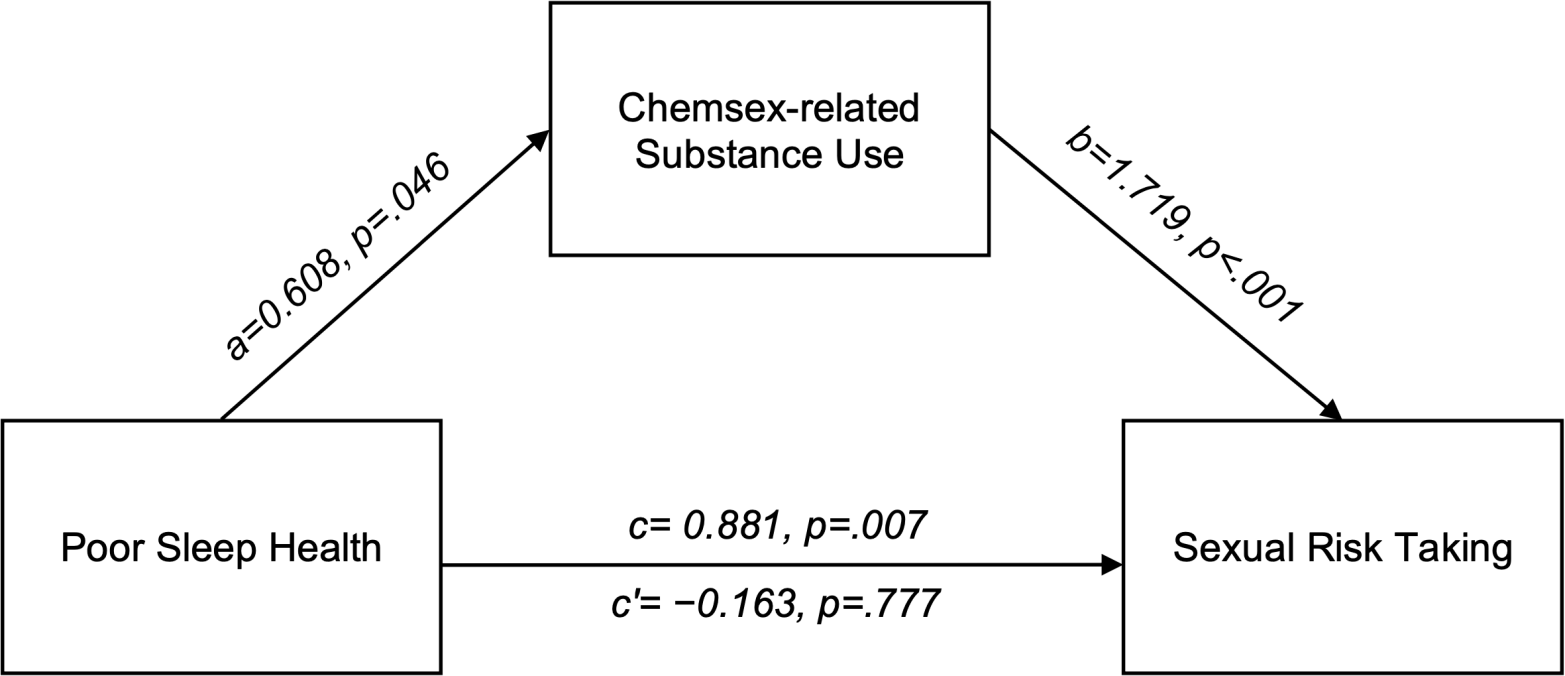
Mediation model of the effect of substance use on the association between poor sleep health and sexual risk taking among LGBTQ+ individuals. Path c represents the total effect of poor sleep on sexual risk-taking. Path a reflects the effect of poor sleep on substance use, while Path b captures the effect of substance use on sexual risk-taking, controlling for poor sleep. Path c’ represents the direct effect of poor sleep on sexual risk-taking after accounting for substance use. Living arrangement, employment, monthly income, gender, and sexual orientation were included as covariates in the model but are not depicted in the figure.

**Figure 1** summarizes the mediation model of the effect of CRSU on the association between PSQ and SRT. The mediation analysis examined the relationship between PSQ and SRT, with CRSU as a mediator. Pathway a (PSQ → CRSU) demonstrated a statistically significant positive association (β=0.608, p=.046). Similarly, Pathway b (CRSU → SRT) showed a significant positive relationship (β=1.719, p<.001). In contrast, the direct effect of PSQ on SRT (Path c) was not statistically significant (β=−0.163, p=.777). The indirect effect of PSQ on SRT through CRSU was significant (β=1.045, p=.047), indicating the mediating role of CRSU. The total effect, combining direct and indirect pathways, was also significant (β=0.881, p=.007), suggesting that the influence of PSQ on SRT primarily operates through its impact on CRSU among LGBTQ+ individuals.

When the relationship between PSQ and SRT was examined with AUDIT-C serving as the mediator, there was no statistically significant indirect pathway (β=0.163, p=.137), suggesting no evidence that alcohol use mediates the relationship between PSQ and SRT. There was also no statistically significant mediation between PSQ and sexual dysfunction via either CRSU (β=-0.173, p=.445) or alcohol use (β=-0.008, p=.942).

To assess the directionality of associations, two alternative mediation models were tested. In the first model, CRSU was specified as the predictor, PSQ as the mediator, and SRT as the outcome. CRSU was significantly associated with worse PSQ (β = 0.247, p = .025), and PSQ was significantly associated with higher SRT (β = 0.218, p = .010); however, the indirect effect of this pathway did not reach statistical significance (β = 0.251, p = .090). In the second model, SRT was specified as the predictor CRSU as the mediator, and PSQ as the outcome. While SRT was strongly associated with CRSU (β = 0.461, p <.001), neither the direct effect on PSQ (β = 0.154, p = .175) nor the indirect effect via CRSU (β = 0.057, p = .152) were statistically significant.

The models in this study were just-identified (degrees of freedom = 0). In such cases, the model necessarily fits the data perfectly by definition, and fit indices are not informative. For this reason, model fit statistics were not reported.

## 4 Discussion

This study highlights significant associations between sleep quality, substance use, and sexual risk taking in an LGBTQ+ cohort in Turkey. PSQ was associated with socioeconomic status, behavioral factors, including substance and alcohol use, SRT, and sexual dysfunction. CRSU was strongly associated with increased SRT and played a mediating role between PSQ and SRT.

80.7% of participants reported PSQ, significantly higher than prior estimates of one-third among LGBTQ+ individuals globally (16, 32). This disparity between our findings and those from studies conducted primarily in Europe and North America may stem from the high rates of discrimination in Turkey (33).

Alcohol and substance use were linked to PSQ, a pattern consistently reported in previous research (16, 34). Among transgender and nonbinary individuals, substance use has been identified as a mediator between discrimination and sleep disturbances (35). Additionally, another study conducted with transgender individuals found that participants used marijuana to cope with sleep difficulties (36).

Although few studies have explored the association between SRT and PSQ, multiple studies linked PSQ to risk-taking behavior (13). A study on MSM linked PSQ to condomless receptive anal intercourse with multiple partners (16). However, existing literature focuses on MSM, likely due to challenges in defining SRT among same-sex women and gender-diverse individuals. Rising HIV incidence in Turkey highlights the public health risks associated with SRT and chemsex. Additionally, the lack of systematic surveillance, compounded by complex political factors, further exacerbates HIV-related public health risks (37).

Research suggests that healthy sleep is crucial for sexual function (15, 21). Conditions such as insomnia and obstructive sleep apnea are strongly associated with male and female sexual dysfunction (38). The interplay between sexual function and sleep quality is complex, as both are influenced by depression, anxiety, and stress (21, 39). However, routine clinical assessments of sexual dysfunction rarely include sleep health. Contrary to expectations, our study found no positive association between substance or alcohol use and sexual dysfunction (40, 41). The substance use patterns of LGBTQ+ individuals may differ from the prolonged use typically associated with sexual dysfunction. Interestingly, substance use in our cohort was linked to lower odds of reporting sexual dysfunction, consistent with literature suggesting that MDMA may enhance sexual function (42).

Chemsex, though lacking a universal definition, generally refers to using substances before or during sex to enhance disinhibition, prolong activity, and increase desire. Common drugs include methamphetamine, cathinones, and GHB/GBL, alongside sildenafil, alcohol, ketamine, and amyl nitrates (43, 44) Shaped by the unique cultural and social factors within the gay community, chemsex is influenced by societal stigmatization. In Turkey, conservative views on LGBTQ+ individuals amplify isolation, shame, and psychological distress (45–47), possibly leading to substance use for disinhibition or escape during sex.

Our results showed that substance and alcohol use is strongly associated with SRT. The use of drugs before sex may impair the individual ability to negotiate safer sex practices (47). A previous study has shown that the prevalence of drug use and chemsex practices are high among patients evaluated for STIs, especially between MSM (44). Advocacy for harm reduction strategies targeting chemsex and other high-risk behaviors could help mitigate these risks.

Our findings suggest that PSQ contributes to SRT indirectly by increasing susceptibility to substance use. According to Duncan et al., chronic PSQ can elevate stress, increasing susceptibility to substance use, which, in turn, may impair decision-making and increase SRT among MSM (16). Official reports from Turkey show drug-related incidents are rising (48), yet data on daily substance use in high-risk groups remain scarce, and no targeted interventions address SRT or chemsex (49). Additionally, a shift toward more harmful substances, such as methamphetamine and GHB, has been observed among MSM (50). These findings suggest that integrating substance use interventions with strategies to improve sleep health may help mitigate the impact of PSQ on SRT in LGBTQ+ individuals. It is crucial to understand that the drugs themselves are not the core issue, but rather the underlying factors that drive their use as a coping mechanism. Addressing this requires culturally competent harm reduction strategies incorporating mental health support and healthcare provider education.

## 5 Strengths and limitations

This study has several limitations that should be considered when interpreting the findings. First, participant recruitment primarily occurred through social media platforms, potentially introducing selection bias. Second, the cross-sectional design of the study makes it susceptible to omitted variable bias, with unmeasured confounding variables potentially influencing the relationships examined in our multivariable and mediation models. Third, all data were collected through self-report measures, making them susceptible to recall bias and social desirability bias. Fourth, the mediation model was just-identified (df = 0). This is a known limitation of saturated mediation models without latent variables. While just-identified models are common in single-mediator models without latent variables, this structure limits the ability to evaluate model adequacy, compare alternative model specifications, or formally test competing theoretical frameworks. Therefore, findings from these models should be interpreted as descriptive rather than confirmatory, and future research should consider more complex or over-identified models to strengthen causal inferences. Lastly, we received extensive feedback from participants regarding the definition of sexual risk-taking for same-sex women and gender-diverse individuals. As a result, three items were omitted from the ISRT to ensure greater inclusivity and relevance. Despite these limitations, this study provides valuable insights into the relationships between sleep health, substance use, and sexual risk-taking among LGBTQ+ individuals, highlighting the need for further research to refine measurement tools and address population-specific risk factors.

## 6 Conclusion

This study provides new insights into how PSQ contributes to SRT among a diverse cohort of LGBTQ+ individuals in Turkey, primarily by increasing susceptibility to substance use. PSQ was prevalent in four out of five participants and strongly associated with indicators of socioeconomic disadvantage, substance and alcohol use, and SRT. Notably, CRSU emerged as a robust mediator between PSQ and SRT. From a public health perspective, these results call for urgent need to develop comprehensive interventions that simultaneously address substance use, sexual health, and sleep quality among high-risk populations. Given the rising incidence of HIV and substance use in Turkey, effective strategies could include awareness campaigns, harm reduction services for chemsex and other forms of substance use, improved access to STI testing and prevention (e.g., PrEP), and specialized LGBTQ+ health training for healthcare providers. Future research should focus on refining measures of sexual risk-taking for non-men, exploring longitudinal data to clarify causal pathways, and examining how discrimination and mental health disparities across gender- and sexual orientation-diverse subgroups may exacerbate poor sleep quality and its consequences.

## Data Availability

The datasets presented in this article are not readily available to protect anonymity and safety of the participants of our study as they represent a small number of participants from a vulnerable group. Requests to access the datasets should be directed to the corresponding author, BS.
