# Supplementary material for: Poor sleep quality indirectly contributes to higher sexual risk-taking by increasing the likelihood of engaging in substance use among LGBTQ+ individuals
Source: Front Psychiatry. 2025 Jun 18;16:1613882. doi: 10.3389/fpsyt.2025.1613882 (PMC12213682; doi:10.3389/fpsyt.2025.1613882)
Supplement: Supplementary file 1 [file Table1.docx]

**Supplementary Table 1** Models of significant factors associated with poor sleep quality among LGBTQ+ individuals

**Variables Model 1, aOR (95% CI) Model 2, aOR (95% CI) Model 3, aOR (95% CI)**

**(N=249) (N=224) (N=138)**

Living arrangement

Dormitory & Friends 2.46 (0.70-8.57) 3.07 (0.77-12.11) 1.87 (0.36-9.70)

Alone 1.76 (0.60-5.20) 1.27 (0.41-3.89) 1.43 (0.33-6.22)

Family 1.46 (0.49-4.31) 1.35 (0.43-4.18) 0.73 (0.17-3.14)

Partner (ref.) - - -

Employment

Student 0.53 (0.17-1.64) 0.67 (0.20-2.14) 1.30 (0.27-6.24)

Unemployed 0.67 (0.16-2.76) 0.60 (0.14-2.56) 1.49 (0.21-10.42)

Employed (ref.) - - -

Monthly income

Unstable **4.64^*^ (1.27-16.95) 4.17^*^ (1.04-16.59)** 1.70 (0.27-10.64)

0-1 MW **10.56^**^ (2.38-46.84) 11.07^**^ (2.28-53.62)** 4.32 (0.60-30.81)

1-2 MW **5.26^**^ (1.53-18.08) 4.03^*^ (1.14-14.16)** 2.85 (0.61-13.39)

2-3 MW 1.95 (0.59-6.45) 1.69 (0.49-5.88) 1.24 (0.26-5.85)

Above 3 MW (ref.) - - -

Gender

TGNB 0.85 (0.36-1.95) 0.82 (0.34-1.95) -

Cisgender (ref.) - - -

Sexual orientation

Asexual perfect divider - -

Heterosexual 0.48 (0.10-2.21) 0.59 (0.11-3.14) -

Bisexual+ 1.46 (0.67-3.18) 1.60 (0.71-3.60) 0.75 (0.26-2.11)

Gay (ref.) - - -

AUDIT-C

Yes **2.86^**^ (1.36-6.00) 2.40^*^ (1.11-5.17) 3.21^*^ (1.17-8.77)**

No (ref.) - - -

Chemsex-related substances^a^

Yes **3.47^*^ (1.03-11.68)** 2.24 (0.64-7.85) 1.59 (0.41-6.21)

No (ref.) - - -

ISRT

0-7 - 1.24 (0.98-1.57) 1.28 (0.96-1.71)

ASEX

Yes - - 2.92 (0.69-12.22)

No (ref.) - - -

Nagelkerke R Square 0.258 0.271 0.232

CI, confidence interval; OR, odds ratio

^a^Includes amphetamine, methylphenidate, methamphetamine, cocaine, MDMA, LSD, GHB, ketamine, magic mushroom, ketamine.

^*^p<0.05, ^**^p<0.01, ^***^p<0.001
